# Reviewer acknowledgement 2013

**DOI:** 10.1186/2049-6958-9-4

**Published:** 2014-01-30

**Authors:** Fernando De Benedetto, Claudio F Donner, Claudio M Sanguinetti

**Affiliations:** 1 SS Annunziata Hospital, Chieti, Italy; 2 Mondo Medico Clinic, Borgomanero, Italy; 3 Quisisana Clinical Center, Rome, Italy

## Abstract

**Contributing reviewers:**

The editors of Multidisciplinary Respiratory Medicine would like to thank all of our reviewers who have contributed to the journal in Volume 8 (2013).

## 

Metin Akgun

Turkey

Hasan Al-Dorzi

Saudi Arabia

Stefano Aliberti

Italy

Nicolino Ambrosino

Italy

Nick Anas

United Kingdom

Filippo Andò

Italy

Sabina Antoniu

Romania

Charlotte Arbelot

France

Abraham Aseffa

Ethiopia

Lone Avnon

Israel

Ericson Bagatin

Brazil

Ahmed BaHammam

Saudi Arabia

Sergio Baldi

Italy

Giovanni Barisione

Italy

Giorgio Bedogni

Italy

Luisa Maria Bellussi

Italy

Giorgio Bertolotti

Italy

Luca Bianchi

Italy

Silvia Bielsa

Spain

Gian Luca Biscione

Italy

Roberto Boffi

Italy

Aina Bolajoko Ajoke

Nigeria

Casper Bollen

Netherlands

John Botha

Austria

Nafiz Bozdemir

Turkey

Alberto Braghiroli

Italy

Fulvio Braido

Italy

Alaa Brik

Egypt

Vito Brusasco

Italy

Claudio Bruschi

Italy

Lilian Calderon-Garciduenas

United States of America

Raffaele Campisi

Italy

Gaetano Caramori

Italy

Stefano Carlone

Italy

Lucio Casali

Italy

Godet Cendrine

France

Piero Ceriana

Italy

Stefania Cerri

Italy

Pascal Chanez

France

Marie Luce Choukroun

France

Nicola Ciancio

Italy

Gaetano Cicchitto

Italy

**Aydin** Ç**iledag**

Turkey

Roberto Copetti

Italy

Lorenzo Corbetta

Italy

Yvon Cormier

Canada

Angelo Guido Corsico

Italy

Peter Alan Coventry

United Kingdom

William Culp

United States of America

Lia D'Ambrosio

Italy

Edvardas Danila

Lithuania

Mehmet Davutoglu

Turkey

Michele De Benedetto

Italy

Francesco De Blasio

Italy

Cinzia de Marco

Italy

Kassu Desta

Ethiopia

Adriano Di Paco

Italy

Orlando Diaz

Chile

Domenico D'Intino

Italy

Claudia Dobler

Australia

Philipp Eickhoff

Austria

Saeid Eslami

Netherlands

Marcia Faganello Mitsuya

Brazil

Bruno Federico

Italy

Giovanni Ferrara

Sweden

Miguel Ferrer

Spain

Serhat Findik

Turkey

Elizabeth Fireman

Israel

Silvia Forte

Italy

Masaki Fujita

Japan

Stefano Gasparini

Italy

Armen Yuri Gasparyan

United Kingdom

Stefania Giedrys-Kalemba

Poland

Hyun Woo Goo

South Korea

Metin Gorguner

Turkey

Jayaprakash Gosalakkal

United States of America

Jesus Guinea

Spain

Hakan Gunen

Turkey

Masayuki Hanaoka

Japan

John Haughney

United Kingdom

Toyoaki Hida

Japan

Peter Howard

United Kingdom

John Hurst

United Kingdom

Aldo Iacono

United States of America

Cristoforo Incorvaia

Italy

Matjaz Jereb

Slovenia

Yi Ji

China

Nurhan Koksal

Turkey

Oğuz Kilinç

Turkey

Hitoshi Kagaya

Japan

Hasan Kahraman

Turkey

Amy Kantar

Italy

Ad Kaptein

Netherlands

Bulent Karadag

Turkey

Sarper Karakucuk

Turkey

Henrike Karim-Kos

Austria

Alicja Kasperska-Zajac

Poland

Servet Kayhan

Turkey

Ebru Kelsaka

Turkey

Monia Khemiri

Tunisia

Emma Kidd

United Kingdom

Vladimir Koblizek

Czech Republic

Rafal Krenke

Poland

Peggy Lai

United States of America

Xuxin Lai

China

Scott Laney

United States of America

Barbara Lanini

Italy

Jörg D. Leuppi

Switzerland

Gennaro Liccardi

Italy

Hongbo Liu

China

Carlo Lombardi

Italy

Bernadette M. Longo

United States of America

Ernesto Losavio

Italy

Maurizio Luisetti

Italy

Ivan Macía

Spain

Roberto Manetti

Italy

Emilio Marangio

Italy

Stefano Marinari

Italy

Salvatore Mariotta

Italy

Dick Markhorst

Netherlands

Rosario Maselli

Italy

Shin Matsuoka

Japan

Manlio Milanese

Italy

Marc Miravitlles

Spain

Gerhard Mostbeck

Austria

Gregory Moullec

Canada

Hiroshi Mukae

Japan

Leon Mutesa

Rwanda

Henry Mwandumba

Malawi

Stefano G. Nardini

Italy

Margherita Neri

Italy

Linda Nici

United States of America

Hervé Normand

France

Elio Novembre

Italy

Chiho Ohbayashi

Japan

Josuel Ora

Italy

**B**ü**lent** Ö**zbay**

Turkey

Nuri Ozkutuk

Turkey

Berkant Özpolat

Turkey

Antonio Palla

Italy

Sunghoon Park

South Korea

Purnima Parthasarathy

India

Franco Pasqua

Italy

Giovacchino Pedicelli

Italy

Riccardo Pela

Italy

Girolamo Pelaia

Italy

Oronzo Penza

Italy

David Phelps

United States of America

Malgorzata Pierzchalska

Poland

Kukla Piotr

Poland

Giuseppe Pirozzi

Italy

Riccardo Pistelli

Italy

Eligio Pizzigallo

Italy

Venerino Poletti

Italy

Francesca Polverino

United States of America

Mario Polverino

Italy

Roberta Polverosi

Italy

Aravind Ponnuswamy

United Kingdom

Paolo Pozzi

Italy

Helmut Prosch

Austria

Ermanno Puxeddu

Italy

Winfried Randerath

Germany

Mohammed Ridai

Morocco

Marcelo Rieder

Brazil

Thomas Ringbaek

Denmark

Carolyn Rochester

United States of America

Andrea Rossi

Italy

Silvia Rossi Ferrario

Italy

Erica Rutten

Netherlands

Eugenio Sabato

Italy

Erkan Melih Şahin

Turkey

Yogesh Saini

United States of America

Yuzuru Sakamoto

Japan

Neus Salord

Spain

Alessandro Sanduzzi

Italy

Antonio Sanna

Italy

Giuseppe Scardina

Italy

Antonio Schindler

Italy

Nicola Scichilone

Italy

Ekrem Seyhan

Turkey

Tania Shakoori

Pakistan

Maurice Sillen

Netherlands

Michael Sims

United States of America

Amit Singh

India

Sunil Sinha

United Kingdom

Carol Smith Hammond

United States of America

Matteo Sofia

Italy

Chandrashekhar Sreeramareddy

Nepal

Penpatra Sripaiboonkij

Thailand

Robert Stockley

United Kingdom

Wei-Juin Su

Taiwan

Jaya Prakash Sugunaraj

United States of America

Lilla Tamasi

Indonesia

Luigi Taranto Montemurro

Italy

Shinji Teramoto

Japan

Alberto Terzi

Italy

Linwei Tian

Hong Kong

Biagio Tinghino

Italy

Ravindranath Tiruvoipati

Australia

Ingrid Louise Titlestad

Denmark

Philip Tonnesen

Denmark

Roberto Torchio

Italy

Yassine Trabelsi

Tunisia

Roberto Tramarin

Italy

Jennifer Trevor

United States of America

Aggelos Tsalkidis

Greece

Sergio Tufik

Brazil

Willy Urassa

Tanzania

Barbara Vagaggini

Italy

David Van Ly

Australia

Floor van Rosse

Netherlands

Carlo Vancheri

Italy

Andrae Vandross

United States of America

Giovanni Viegi

Italy

Rigoletta Vincenti

Italy

Michele Vitacca

Italy

Claus Vogelmeier

Germany

Kathleen Walsh

United States of America

Insu Yilmaz

Turkey

Dongfang Yu

United States of America

Jian Yu

United States of America

Pietro Zanon

Italy

Annalise Zemlin

South Africa

Guo-qing Zheng

China

Jan Zielinski

Poland

